# The use of urea for the treatment of onychomycosis: a systematic review

**DOI:** 10.1186/s13047-019-0332-3

**Published:** 2019-04-11

**Authors:** S. Dars, H. A. Banwell, L. Matricciani

**Affiliations:** 1 Alliance for Research in Exercise, Nutrition and Activity (ARENA), University of South Australia, Adelaide, 5001 Australia; 2 International Centre for Allied Health Evidence, School of Health Sciences, University of South Australia, City East, Adelaide, SA 5001 Australia

**Keywords:** Onychomycosis, Urea, Treatment, Systematic review, Fungal nail

## Abstract

**Background:**

Onychomycosis, a fungal infection affecting the nail plate, is a common condition often requiring prolonged treatment regimens, with low success rates. Urea is one treatment option, which is thought to improve the efficacy of topical and oral antifungal agents. Despite a theoretical basis for the use of urea for the treatment of onychomycosis, the evidence-base for this treatment has not been systematically reviewed.

**Aim:**

The purpose of this study was to conduct a systematic literature review to determine the efficacy and safety of urea as a monotherapy and as adjunct therapy, compared to other treatment regimens for onychomycosis.

**Method:**

A systematic literature search of ten electronic databases was conducted. Only studies that used microscopy and culture or other validated laboratory-based testing method to confirm the presence of a fungal infection before treatment were included. The outcome measures assessed were efficacy (defined in terms of mycological, clinical and complete cure) and safety (defined as self-reported adverse events).

**Results:**

The systematic search yielded 560 unique studies for review. Of these, only six were eligible for inclusion. All studies were observed to have methodological concerns, most studies consisted of small sample sizes and were difficult to compare given heterogeneity in outcome measures and follow-up time. Despite this, a trend was observed to suggest that urea, when added to topical or oral antifungal treatment regimens, improved efficacy of the treatment.

**Conclusion:**

This review suggests that topical urea, as an adjunct to topical and oral antifungal treatment regimens, may improve the efficacy of treatment. However, further research is needed.

**Electronic supplementary material:**

The online version of this article (10.1186/s13047-019-0332-3) contains supplementary material, which is available to authorized users.

## Background

Onychomycosis, a fungal infection of the nail plate, is a common dermatological condition frequently observed in clinical practice [1–4]. Estimates suggest approximately 5.5% of people are affected world-wide, with a greater incidence among elderly and immunocompromised individuals, as well as people with comorbidities, such as peripheral arterial disease and diabetes [2, 5–8]. Primarily caused by dermatophyte infection (specifically Trichophyton, Epidermophyton or Microsporum) or non-dermatophyte moulds or yeasts (e.G. *candida*) [9], onychomycosis often presents as nail dystrophy, discoloration and onycholysis, with or without strong odour [3–5]. The condition may be painful [1], cosmetically displeasing [4] and negatively affect self-esteem and quality of life [5, 7], as well as result in injury to adjacent skin and infections [5, 7]. Effective and safe treatment is therefore essential.

Several traditional treatment options are available for onychomycosis, including topical antifungal regimens, systemic oral medications, as well as emerging therapies, such as laser, iontophoresis, UV light and photodynamic therapy [6–8, 10]. Whilst emerging therapies are gaining popularity, they are often expensive to set up and there is little evidence to support their use in onychomycosis [11, 12]. Traditional treatment choice often depends on the type and severity of the infection, as well as patient comorbidities [3, 6]. There is a general consensus that topical treatments may be effective in mild to moderate cases, where less than 50% of the nail plate is affected and in the absence of nail matrix involvement [3, 10]. Oral medications are often reserved for more severe cases [6] and may be associated with an increased risk of hepatotoxicity and at times, contraindicated in individuals with affected kidney or cardiac function [6].

Despite the availability of different treatment options, complete cure of onychomycosis is challenging to achieve [1, 5–8, 11, 13]. Two Cochrane reviews have examined the evidence for oral [14] and topical [10] treatment intervention for onychomycosis. Unlike the body of evidence examining the efficacy of oral medication [14], few (mostly small) studies have investigated the efficacy of topical treatments for onychomycosis [10]. Despite this disparity, early and effective treatment with a topical antifungal agent is often preferred, particularly for those most at risk of onychomycosis, who are often unable to tolerate the side-effects of oral medication. Topical treatments, however, often require prolonged treatment regimens [at least twelve months) and have low success rates [10]. Efforts that improve the efficacy of topical antifungal treatments are therefore of interest.

Chemical nail avulsion with topical urea cream has been suggested to improve the efficacy of topical antifungal treatments by improving penetration and bioavailability of topical agents [4, 6, 7, 10]. Urea, in concentrations over approximately 30%, is considered a keratolytic agent [15] that softens and hydrates the nail plate by denaturing the nail keratin and thus enhancing the drug penetration and promoting the avulsion of affected nails [3, 4, 7]. Urea has long been used in dermatology and podiatry [15] for the treatment of onychomycosis [16–21]. Despite the theoretical basis to support the use of urea in the treatment of onychomycosis, the efficacy of urea as a monotherapy and an adjunct treatment remains unclear.

The purpose of this systematic literature review was to determine the evidence for the use of urea for the treatment of onychomycosis. Specifically, the efficacy and safety of urea as monotherapy and adjunct therapy, compared to standard and traditional treatment regimens.

## Methods

A systematic literature review was undertaken to identify all studies that examine the efficacy and/or safety of urea in the treatment of onychomycosis. Specifically, urea as a monotherapy and as an adjunct therapy, compared to standard and traditional treatment regimens.

### Systematic search

This review was conducted and reported in line with the Preferred Reporting Items for Systematic Reviews and Meta-Analyses (PRISMA) statement [22]. Ten electronic databases were searched: Cochrane Central Register of Controlled Trials (CENTRAL), Ovid Medline, Allied and Complementary Medicine Database (AMED), Ovid Embase, Ovid Emcare, The Cumulative Index to Nursing and Allied Health Literature (CINAHL), Scopus, PubMed, Web of Science and Clinical Trials.gov.

The following search terms were used with truncation and MESH headings where relevant: Onychomycosis, tinea unguium, mycotic nail*, fung*al nail, nail fung*us, fung*al nail infection, dystroph*ic nails, onycholy*sis, dermatophyte*es, trichophyt*on rubrum, ringworm, urea, topical drug and topical administration. The search was not limited by date or language and the last search was conducted in mid-December 2017. Secondary search was performed on reference lists, cited by similar or recommended articles sections in different databases.

All search results were pooled and duplicates were removed. Two independent reviewers (SD and HB) screened titles and abstracts for eligibility (criteria described below) before reviewing the full texts. Any disagreements were resolved by discussion with a third reviewer (LM).

### Studies included for review

To maximise the potential for data capture, all forms of primary research design were considered, including randomised controlled trials (RCTs), clinical control trials (CCTs), quasi-experimental, pre-post cohort studies and case studies. The eligibility criteria for the population-intervention-comparator-outcome (PICO) is outlined below.

### Population

Studies were included if the diagnosis of onychomycosis (of fingernails or toenails) was established using microscopy and culture, or alternative laboratory-based tests (e.g. polymerase chain reaction (PCR) testing or periodic acid–Schiff (PAS) staining), to confirm the presence of a fungal infection before treatment was commenced.

### Intervention

Studies were included if the intervention was urea either as monotherapy or in combination with an antifungal agent where the effects of urea alone were determinable. The studies using urea as a control treatment were also included.

### Comparator

The acceptable comparators were alternate interventions (topical or systemic anti-fungal agent or different urea-based treatment regimen). Studies that examine urea as a monotherapy, without a comparator were also considered for inclusion.

### Outcome

The two main outcomes of interest were efficacy and safety. Efficacy was defined as clinical, mycological or complete cure (both clinical and mycological). Safety was defined as any reported adverse effects, such as irritation, erythema or itching.

### Data extraction and analysis

All studies included for review were read in full by two independent reviewers (SD and LM). Data extraction involved recording details of the study design and level of evidence, sample characteristics, intervention/s, comparator/s and outcome measure of interest (efficacy and safety). To determine the level of evidence of included studies, the Intervention category of the Australian National Health and Medical Research Council’s (NHMRC) evidence hierarchy was used [23]. Data were extracted by two independent reviewers (SD and LM) using Covidence® (Veritas health innovation LTD 2018), with any disputes resolved through discussion with a third reviewer (HB).

The NHMRC FORM methodology [24] was used in the interpretation of findings and the implications for clinical practice. Previous systematic reviews have used this framework successfully [25–27]. The framework consists of five main components: 1) evidence base (level on evidence hierarchy); 2) consistency; 3) clinical impact; 4) generalizability; and 5) applicability to the Australian health care setting. This last component was not used for this systematic review due to its international focus. Data extracted from included studies were examined descriptively.

### Critical appraisal of methodological quality

The methodological quality of studies included for review was assessed using the McMaster Critical Review Form for Quantitative Studies [28, 29]. This critical appraisal tool assessed eight main components including: study purpose; literature review; study design (all experimental designs); sample (participants’ description, size justification, ethics and consent); outcomes (reliability and validity, outcome areas and measures used); intervention (description, contamination and co-intervention); results (statistical and clinical significance, analysis methods and drop outs) and conclusion with implications to practice (limitations and biases).

To suit this review, the McMaster Critical Review Form for Quantitative Studies was modified to include questions on the randomisation of groups where relevant, and the reliability of the assessment methods used to establish the diagnosis of onychomycosis. The individual components were rated as ‘yes’, ‘no’, ‘not-addressed’ or ‘not applicable (NA)’. A score of ‘1’ was given to ‘yes’ and ‘0’ to ‘no and not-addressed’ while if ‘NA’ category applied than the total scoring was changed accordingly. The total score depended on the research design and relevant components with the maximum score being 17 (Additional file 1).

Two reviewers (SD and LM) independently assessed the methodological quality of the included studies and any disputes were resolved through discussion.

## Results

**Fig. 1:**
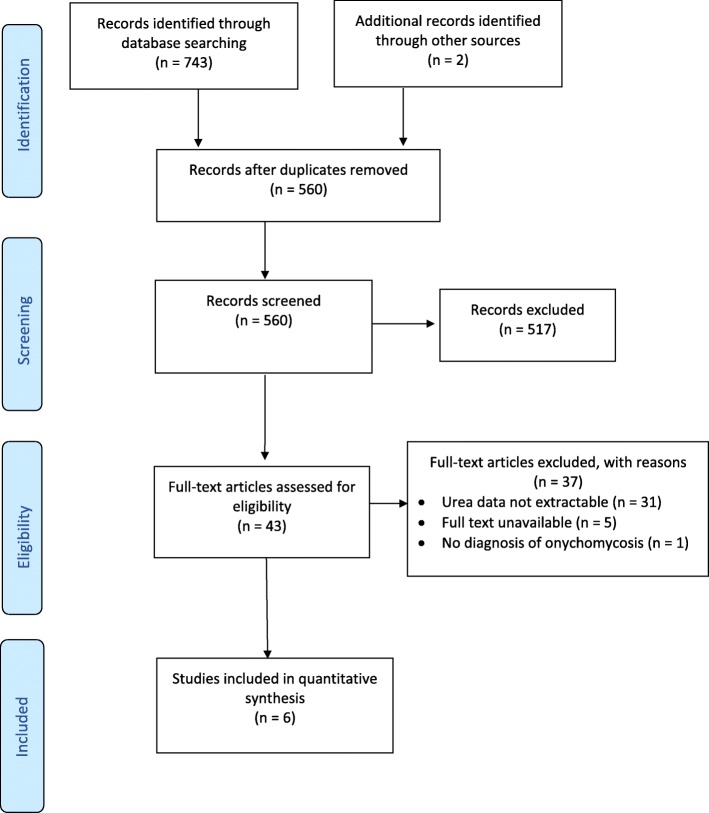
PRISMA flow chart of selection criteria

The systematic search strategy identified 560 unique titles. Of these, six studies met the eligibility criteria. The study selection process is outlined by PRISMA flowchart in Fig. 1.

### Studies included for review

Table 1 presents a summary of each of the studies included for review. Study sample sizes ranging from 10 to 114 participants, covering adult participants, predominantly males, within a wide age range (19 to 78 years).

**Table 1:** Study and participants characteristics

Study	N	Mean age + SD (range)years	Gender (M = Males, F=Females)	Pathogens identified (n)	Type of Onychomycosis (n)	Intervention	Comparator/control	Intervention frequency	Follow up times
Bassiri -Jahromi et al. 2012 [30]	70	50.4 (29–78)	42 M 28 F	T.Rubrum (52) T.Mentagrophytes (13) T.Verrucosum (1)	DLSO (57) PSO (13)	40% urea with 1% fluconazole (urea in combination)	1% fluconazole alone	Once daily for six months	First = monthly until 6 months After Tx – 2,4 and 6 months
Lahfa et al. 2013 [31]	105	54.3 ± 14.9	66 M 39 F	NA	NA	40% urea, then bifonazole cream for 8 weeks (urea as adjunct)	1% bifonazole + 40% urea cream, then bifonazole cream for 8 weeks (urea in combination)	Once daily for 3 weeks	First = 21 days Second = 4 weeks
Bunyaratavej at al. 2016 [32]	53	67.8 ± 10.7	33 M 20 F	NA	DLSO (52) SWO (1)	40% urea	5% amorolfine nail lacquer	Once daily	Every 2 months until complete cure After Tx – every 6 months for 2 years
Fraki et al. 1997 [33]	114	44 (19–70)	63 M 51 F	T.Rubrum (112) T.Mentagrophytes (1) T.Tonsurans (1)	NA	40% urea, then 150 mg fluconazole (urea as adjunct)	150 mg fluconazole oral	Once only	Every month until cure or 12 months After Tx – 1,3 and 6 months
Escalante et al. 2013 [34]	55	NR	19 M 36 F	T.Rubrum (26) T.Mentagrophytes (2)	TDO (21)	40% urea	Group 1 - 250 mg oral terbinafine Group 2 – 250 mg oral terbinafine + 40% urea cream (urea in combination)	Once nightly for 4 weeks	First = 12 weeks After Tx – 12 weeks
Baran and Tosti 2002 [35]	10	22–65	8 M 2 F	T.Rubrum (7) Candida (1) Other pathogens (2)	DLSO (8) TDO (2)	40% urea	NA	Twice daily for one week	7 days

Pathogens identified = Trichophyton Rubrum (T.Rubrum), Trichophyton Mentagrophytes (T.Mentagrophytes), Trichophyton Tonsuran (T.Tonsuran) and Trichophyton Verrucosum (T.Verrucosum). Types of Onychomycosis = Distal and lateral subungual onychomycosis (DLSO), Proximal subungual onychomycosis (PSO), Superficial White Onychomycosis (SWO) and Total Dystrophic Onychomycosis (TDO)

*NR* not reported, *Tx* treatment

Table 2 presents the methodological quality of studies, as rated by a modified McMaster Critical Review Form for Quantitative Studies [28]. As presented, only two studies [30, 31] provided Level II (randomised controlled trial (RCT)) evidence. The main methodological concerns were: lack of justification of the sample size (only one study did the power calculation [31], lack of psychometrically robust outcome measures (OMs) (validity and reliability recorded only in one study [31]), failure to avoid contamination and co-intervention and lack in reporting statistical and clinical significance of the results (four studies reported statistical significance [31–34] and none reported clinical significance). Furthermore, the randomisation methods were not appropriate where groups were randomised [30, 33].

**Table 2:** NHMRC levels of evidence and modified McMaster results of methodological quality

Study	NHMRC level and study design	Items on modified McMaster critical review form																	Raw score and %
		1	2	3a	3b	3c	3d	3e	4a	4b	5a	5b	5c	6a	6b	6c	6d	7	
Bassiri -Jahromi et al. 2012 [30]	Level II-RCT	Y	N	Y	N	Y	N	Y	N	N	Y	N	Y	N	Y	N	Y	N	8/17 47.06%
Lahfa et al. 2013 [31]	Level II-RCT	Y	Y	Y	Y	Y	Y	Y	N	N	Y	Y	Y	Y	Y	N	Y	Y	14/17 82.35%
Bunyaratavej at al. 2016 [32]	Level III-2 Case-control	Y	Y	Y	N	N	NA	Y	N	N	Y	Y	Y	Y	Y	N	NA	Y	10/15 66.60%
Fraki et al. 1997 [33]	Level III-3 Comparative study without controls	Y	Y	Y	N	Y	N	Y	N	N	Y	Y	N	Y	N	N	Y	Y	10/17 58.82%
Escalante et al. 2013 [34]	Level III-3 Comparative study without controls	Y	Y	Y	N	Y	Y	Y	N	N	Y	Y	N	Y	Y	N	Y	Y	12/17 70.59%
Baran and Tosti 2002 [35]	Level IV Case-series	N	Y	Y	N	NA	NA	Y	N	N	N	N	N	N	N	N	NA	Y	4/14 28.57%

McMaster items to be scored: 1. Was the purpose stated clearly?; 2. Was relevant background literature reviewed?; 3a. Was the sample described in detail?; 3b. Was sample size justified?; 3c. Were the groups randomised?; 3d. Was randomising appropriately done?; 3e. Was the diagnostic method for onychomycosis appropriate?; 4a. Were the outcome measures reliable?; 4b. Were the outcome measures valid?; 5a. Intervention was described in detail?; 5b. Contamination was avoided?; 5c. Cointervention was avoided?; 6a. Results were reported in terms of statistical significance?; 6b. Were the analysis method/s appropriate?; 6c. Clinical importance was reported?; 6d. Drop-outs were reported?; and 7. Conclusions were appropriate given study methods and results?. Y = yes, N = No, NA = not applicable

### Interventions and controls

All included studies used 40% urea as either an intervention or control, no other percentage of urea was reviewed (Tables 3 & 4). Three studies examined urea as a monotherapy (urea alone), [32, 34, 35], two studies reviewed urea as an adjunct prior to treatment with other anti-fungal medicaments (urea as adjunct), [31, 33], three studies investigated urea used concurrently with other anti-fungal medicaments (urea in combination), [30, 31, 34].

**Table 3:** Outcome domains and measures

	Bassiri -Jahromi et al. 2012 [30]	Lahfa et al. 2013 [31]	Bunyaratavej at al. 2016 [32]	Fraki et al. 1997 [33]	Escalante et al. 2013 [34]	Baran and Tosti 2002 [35]
Clinical improvement	Photographs	Judged by investigator	(Scoring Clinical Index for Onychomycosis (SCIO) score). Decrease in thickness of subungual hyperkeratosis from the original untreated nail			
Clinical cure		Judged by investigator (> 90% clinical improvement)	Scoring Clinical Index for onychomycosis (SCIO) score (> 90% clinical improvement)	Visual inspection, investigator judgement (> 90% clinical improvement)	Nail dystrophy, thickness and a photographic record	
Mycological cure	Fungal culture	Microscopy and fungal culture	Potassium hydroxide and fungal cultures	Microscopy and fungal culture	Potassium hydroxide and fungal cultures	
Complete cure		Mycological cure + clinical cure	Mycological cure + clinical cure			
Adverse events	Participant-reported	Investigator assessment of erythema, irritation, pruritus, desquamation, and patient self-reporting of a burning sensation of the skin surrounding the treated nail (4-point scales)			Participant- reported and clinician driven visual inspection	Participant-reported and clinician driven visual inspection

**Table 4:** Efficacy of urea for the treatment of onychomycosis

	Medicament/s	Chemical avulsion	Clinical improvement	Clinical cure	Mycological cure	Complete cure (clinical + mycological)	Adverse effects
Bassiri -Jahromi et al. 2012 [30]	40% urea with 1% fluconazole cream (urea in combination)		77.1%		82.8%		1 (of 70) reported mild irritation
	1% fluconazole cream		68%		62.8%		
Lahfa et al. 2013 [31]	40% urea then 1% bifonazole cream (urea as adjunct)	86.3%	40.4%		42.6%	27.7%	94.1% tolerability
	40% urea with 1% bifonazole cream (urea in combination)	60.8%	29.2%		58.3%	20.8%	
Bunyaratavej at al. 2016 [32]	40% urea		48%	20%	32%	20%	NR
	5% amorolfine lacquer		85.7%	50%	89.3%	50%	
Fraki et al. 1997 [33]	40% urea for 1 week then 150 mg fluconazole (urea as adjunct)			71%	69%		
	150 mg fluconazole			51%	84%		
Escalante et al. 2013 [34]	40% urea			7%	8.3%		3 of 12 reported periungual maceration. 1 of 12 reported onychocryptosis
	250 mg terbinafine			43%	8%		
	40% urea and 250 mg terbinafine (urea in combination)			50%	57%		
Baran and Tosti 2002 [35]	40% urea	100%					Nil adverse effects reported

Comparators were other topical antifungals (1% fluconazole, 1% bifonazole and 5% amorolfine), [30–32], and oral anti-fungal agents (150 mg fluconazole and 250 mg terbinafine), [34, 35], (Table 3).

### Outcome measures

A range of outcome measures were utilised to evaluate the efficacy and safety of treatment interventions for onychomycosis. Treatment efficacy was considered in terms of mycological, clinical and complete improvement or cure, while safety was considered in terms of patient- or clinician-report. Overall, three studies [30–32] examined clinical improvement (improvement observed visual inspection), four studies [31–34] reported clinical cure (> 90% clinical improvement) and five studies [30–34] reported mycological cure. Two studies [31, 32] assessed complete cure (defined as clinical and mycological cure). Adverse events were reported in four studies [30, 31, 34, 35]. Table 3 provides a summary of the outcome measures examined across studies.

### Efficacy of urea for the treatment of onychomycosis

Table 4 details the efficacy of urea when used alone, as an adjunct or in combination with other medicaments.

### Clinical improvement/cure

Three studies reported on clinical improvement by using different measures like photographs [30], visual inspection by investigator [31] and Scoring Clinical Index for Onychomycosis (SCIO) [32]. Bunyaratavej et al. 2016 described SCIO scoring to range from 1 to 30 and a higher score indicated higher severity of onychomycosis. Moreover, SCIO scoring consists of clinical and growth components encompassing the location (which digit), area of infection on nail plate, thickness of subungual hyperkeratosis and age of the patient. The validity and reliability of the index however was not identified.

Two [30, 31] of the three [30–32] studies used urea as an intervention and reported greater clinical improvement when compared to control groups. One of the studies used urea as a control and reported less clinical improvement (48%) when compared to the intervention (amorolfine) group (85.7%) [32] (Table 4).

Clinical cure was identified by studies as > 90% clinical improvement. The outcome measures used to identify clinical cure included investigators’ judgement [31, 33, 34], photographs [34] and Scoring Clinical Index for onychomycosis (SCIO) score [32].

### Mycological cure

**Fig. 2:**
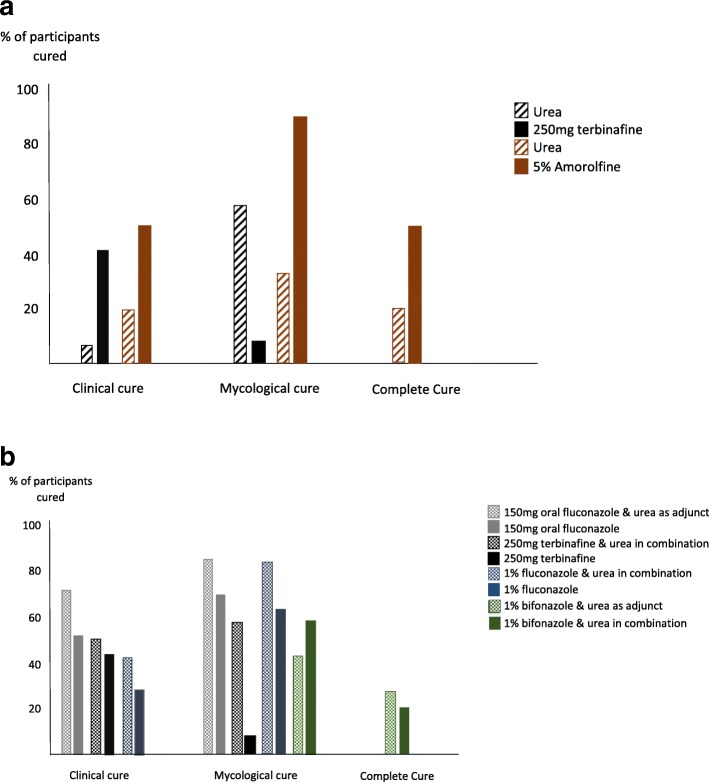
**a** Studies that determined the efficacy of urea as monotherapy compared to standard treatment regimens. **b** Studies that determined the efficacy of urea when used as an adjunct or in combination to standard treatment regimens

Five studies identified mycological cure using microscopy and fungal cultures [30–34]. This was the most common outcome measured with the consistency in the outcome measures used. However, all studies failed to comment on the validity and reliability of the microscopy and fungal cultures. The mycological cure achieved varied from 8.3 to 82.8% when using either urea on its own, urea nail removal before starting topical antifungal cream or urea in combination with a topical antifungal (Table 4, Fig. 2a & b).

### Complete cure

Complete cure, as a combination of clinical and mycological cure, being the most desired outcome clinically was reported by two studies [31, 32]. A complete cure was reported in 27.7% [31] and 20% [32] in urea group compared to 20.8% in urea-bifonazole and 50% in amorolfine group, respectively.

### Summary of cure results

Across the areas of clinical, mycological and complete cure, urea was found less effective than oral terbinafine [34] and topical amorolfine [32], when used alone (Table 4, Fig. 2a). Statistically significant improvements in the efficacy of treatment was reported in three studies when urea was used as an adjunct or in combination with topical or oral antifungal medicaments [30, 33, 34], (Table 4, Fig. 2b). When comparisons were made between urea as an adjunct prior to the application of 1% bifonazole cream and urea in combination with 1% bifonazole cream [31], the reported mycological cure was higher for the combination treatment (58.3 vs 42.6% respectively), but the complete cure was higher when used as an adjunct (27.7 vs 20.8% respectively), albeit not statistically significant in both instances (Table 4, Fig. 2b). Although a limited number of studies were identified, two [30, 31] presented results of RCT study design, representing the higher level of evidence. These RCT studies indicated effectiveness of urea when used with other medicaments for clinical improvement and mycological cure [30, 31] and complete cure [31] respectively.

### Safety of urea for the treatment of onychomycosis

A total of four studies [30, 31, 34, 35] reported the safety of urea for the treatment of onychomycosis. Of these, three [30, 31, 35] studies reported mild to moderate adverse events including: periungual maceration in 25% of participants (3 of 12) of a comparative study [34]; redness and tingling in less than 1% of participants (1 of 70) in an RCT study [30], and; in an alternative RCT [31], 94.1% of participants reported local tolerability (99 of 105) with urea treatment. This last RCT also reported 30% of participants were ‘very’ satisfied with the overall efficacy of urea treatment upon completion of the study [31].

### NHMRC FORM framework

The analysis of results using NHMRC FORM framework is summarised in Table 5. Given the overall body of evidence was limited in size and has methodological flaws, therefore, implementation of recommendations should be undertaken with caution.

**Table 5:** NHMRC FORM framework

Component	Grade	Comments
Evidence base	C–Satisfactory *One or two level III studies with a low risk of bias, or level I or II studies with a moderate risk of bias*	Quantity: Total of 6 studies Level II: 2 studies; Level III-2: 1 study; Level III-3: 2 studies Level IV: 1 study;
Consistency	C–Satisfactory *Some inconsistency*	Multiple study designs All included studies used 40% urea Good consistency with diagnostic criteria Varied outcomes but some consistency
Clinical impact	D–Poor *Slight or restricted*	While four studies reported statistical significance, clinical significance was not reported at all.
Generalisability	B–Good *Population(s) studied in body of evidence is/are similar to the target population*	Population studied in the evidence base is similar to the target population; Age range: 22–78 years Consistent diagnostic criteria and concentration of urea used
Grade of recommendations	C–Satisfactory *Body of evidence provides some support for recommendation(s) but care should be taken in its application*	Overall, most studies are of moderate methodological quality; There was consistency noticed in the diagnostic criteria, intervention/control and outcomes measured. The lack of long-term follow-up existed.

## Discussion

This study reviewed the efficacy and safety of urea for the treatment of onychomycosis. Only a small body of literature, consisting of six studies, were found to investigate urea for management of onychomycosis. These studies seem to suggest urea as an adjunct or in combination with standard oral and topical treatment regimens, improves the efficacy of treatment, while remaining reasonably safe. However, due to the small sample sizes of the included studies, inconsistencies in protocols and variations in comparators and outcome measures, caution is required in interpreting these findings.

On review of the available data, several considerations require attention. Firstly, generalisability of study results is limited by small sample sizes and wide age range examined. Secondly, although all studies investigated mycosis in adults, the pathogen, type of infection and percentage of nail involvement varied or was not reported (Table 1). Thirdly, assessment periods and follow-up times varied considerably across studies. The majority of the studies [30, 31, 34, 35] followed participants for 6 months or less, with one concluding 6 months following ‘complete cure’ [33] and one continuing for 2 years [32]. Fingernails grow faster than toenails (approximately 3.5 vs. 1.6 mm/month in young adults) [36], however ageing and the presence of disease is known to slow growth [37] suggesting a 6 month period may not be adequate. At the very least, these short time periods do not allow for adequate identification of infection relapse. Furthermore, the majority of the studies focused on mycological cure, which is defined as a negative nail culture and microscopy results [38]. Whereas a complete cure, which considers the mycological outcome as well as visual improvements in nail appearance (known as clinical cure) was only investigated twice [31, 32]. In both studies complete cure rates were reduced in comparison to mycological cure.

Given the concerns identified in the literature regarding the penetration and bioavailability of topical anti-fungal agents in the diseased nail plate [4, 7], one use of urea can be partial or complete nail avulsion prior to standard topical anti-fungal treatment. Two studies reported successful chemical avulsion of clinical infected nail plate using 40% urea [31, 35]. Lahfa et al. 2013 [31], found statistically significant difference in nail avulsion rate in urea group when compared to control group, 86.3% vs 60.8% (*p* = 0.028) and reported use of urea once daily for 3 weeks for nail avulsion before a topical antifungal (bifonazole) therapy for 8 weeks. Baran and Tosti 2002 [35] reported 100% successful nail avulsion after application of urea with occlusion twice daily for 1 week with no antifungal treatment after nail avulsion. However, there was no control or comparator used. From a podiatric practice standpoint, this suggests urea could be a useful adjunct service in the management of fungal nail infections and may have potential to be an alternative method of nail remove where mechanical debridement or surgical intervention is contraindicated. More research is required, however, to determine the efficacy and safety of urea as a monotherapy.

Despite limited evidence on the efficacy and safety of urea for the treatment for onychomycosis, available studies seem to suggest that urea as an adjunct therapy to standard treatment regimens, improves the efficacy, while remaining safe. This is an important finding that warrants further investigation, particularly given that standard treatment regimens often involve prolonged treatment times with poor success rates [6, 8, 10]. Given that many patients at risk of onychomycosis and at risk of associated complications are resistive to standard topical treatments or ineligible for oral medications, urea may be a promising adjunct to traditional treatment options. Further, urea appears cost effective, is accessible and will not require invasive pre-test clearance. Topical urea is therefore potentially a feasible option for podiatry-guided treatments of onychomycosis. Further studies are however needed to better understand the effects of different urea concentrations, application techniques and treatment regimens.

## Conclusion

This review identifies a limited number of studies, of varying methodological quality, that examine the efficacy and safety of urea for the treatment of onychomycosis. While definitive conclusions cannot be drawn from this review, available studies suggest urea, as an adjunct to standard treatment regimens, may improve the efficacy of treatment. Urea alone, however, does not appear superior to standard treatments. Further research is needed to determine the efficacy and safety of urea as an adjunct to traditional onychomycosis treatment regimens.

## Additional file

Additional file 1: Modified McMaster tool (DOCX 17 kb)

## Abbreviations

SCIO: Scoring Clinical Index for Onychomycosis
