# Supplementary material for: The use of urea for the treatment of onychomycosis: a systematic review
Source: J Foot Ankle Res. 2019 Apr 11;12:22. doi: 10.1186/s13047-019-0332-3 (PMC6458736; doi:10.1186/s13047-019-0332-3)
Supplement: Supplementary file 1 — Modified McMaster tool (DOCX 17 kb) [file 13047_2019_332_MOESM1_ESM.docx]

## **Appendix 1 – Modified McMaster tool**

Modified McMaster Critical Review form

Fields in ‘red text’ have been added. Maximum score = 17 (depending on the type of study, for example, if study was not a randomised controlled trial then randomisation components were marked as NA thus changing the total score).

| **Assessment Components** | **Yes** | **No** | **Not addressed** | **Not Applicable** |
| --- | --- | --- | --- | --- |
| **Study Purpose** |  |  |  |  |
| Was the purpose of the study clearly stated? | 1 |  |  |  |
| **Literature Review** |  |  |  |  |
| Was relevant background literature reviewed? | 2 |  |  |  |
| **Study Design** |  |  |  |  |
| RCT |  |  |  |  |
| cohort |  |  |  |  |
| single case design |  |  |  |  |
| before and after |  |  |  |  |
| case control |  |  |  |  |
| cross sectional |  |  |  |  |
| case study |  |  |  |  |
| **Sample** | **Yes** | **No** | **Not addressed** | **Not Applicable** |
| Was the sample described in detail? | 3 |  |  |  |
| Was sample size justified? | 4 |  |  |  |
| Were the groups randomised? | 5 |  |  |  |
| Was randomising appropriately done? | 6 |  |  |  |
| Was mycology identification method reported? | 7 |  |  |  |
| **Outcomes** | **Yes** | **No** | **Not addressed** | **Not Applicable** |
| Were the outcome measures reliable? | 8 |  |  |  |
| Were the outcome measures valid? | 9 |  |  |  |
| **Intervention** |  |  |  |  |
| Intervention was described in detail? | 10 |  |  |  |
| Contamination was avoided? | 11 |  |  |  |
| Cointervention was avoided? | 12 |  |  |  |
| **Results** | **Yes** | **No** | **Not addressed** | **Not Applicable** |
| Results were reported in terms of statistical significance | 13 |  |  |  |
| Were the analysis method/s appropriate? | 14 |  |  |  |
| Clinical importance was reported? | 15 |  |  |  |
| Drop-outs were reported? | 16 |  |  |  |
| **Conclusions and clinical implications** | **Yes** | **No** | **Not addressed** | **Not Applicable** |
| Conclusions were appropriate given study methods and results? | 17 |  |  |  |
